# Effects of omega-3 fatty acids on arterial stiffness in patients with hypertension: a randomized pilot study

**DOI:** 10.1186/s12952-015-0040-x

**Published:** 2015-12-02

**Authors:** Mori J. Krantz, Edward P. Havranek, Rocio I. Pereira, Brenda Beaty, Philip S. Mehler, Carlin S. Long

**Affiliations:** Cardiology Division, Denver Health Medical Center, 777 Bannock St., MC0960, Denver, CO 80204 USA; Department of Medicine, University of Colorado School of Medicine, 13001 E 17th Pl., Aurora, CO 80045 USA; Colorado Health Outcomes Program, University of Colorado School of Medicine, 13199 E. Montview Blvd. Suite 300, Aurora, CO 80045 USA

**Keywords:** Pulse wave velocity, LpPLA2, Hypertension, Latino, C-reactive protein

## Abstract

**Background:**

Omega–3 fatty acids prevent cardiovascular disease (CVD) events in patients with myocardial infarction or heart failure. Benefits in patients without overt CVD have not been demonstrated, though most studies did not use treatment doses (3.36 g) of omega-3 fatty acids. Arterial stiffness measured by pulse wave velocity (PWV) predicts CVD events independent of standard risk factors. However, no therapy has been shown to reduce PWV in a blood pressure-independent manner. We assessed the effects of esterified omega–3 fatty acids on PWV and serum markers of inflammation among patients with hypertension.

**Design and methods:**

We performed a prospective, randomized; double-blinded pilot study of omega-3 fatty acids among 62 patients in an urban, safety net hospital. Patients received 3.36 g of omega–3 fatty acids vs. matched placebo daily for 3-months. The principal outcome measure was change in brachial-ankle PWV. Serum inflammatory markers associated with CVD risk were also assessed.

**Results:**

The majority (71 %) were of Latino ethnicity. After 3-months, mean change in arterial PWV among omega-3 and placebo groups was −97 cm/s vs. −33 cm/s respectively (*p* = 0.36 for difference, after multivariate adjustment for baseline age, systolic blood pressure, and serum adiponectin). Non-significant reductions in lipoprotein-associated phospholipase A2 (LpPLA2) mass and high sensitivity C-reactive protein (hsCRP) relative to placebo were also observed (*p* = 0.08, and 0.21, respectively).

**Conclusion:**

High-dose omega-3 fatty acids did not reduce arterial PWV or markers of inflammation among patients within a Latino-predominant population with hypertension.

**Clinical trial registration:**

NCT00935766, registered July 8 2009.

## Background

The effects of omega-3 fatty acids on cardiovascular disease (CVD) outcomes have been mixed. Prospective randomized trials have previously demonstrated reductions in CVD events among patients with myocardial infarction [1] and heart failure [2]. However, no benefits have been reported among patients without documented CVD [3, 4]. It is unclear if the relatively low dose (1g) of omega-3 fatty acids utilized in these trials contributed to the null effect since the therapeutic dosage for hypertriglyceridemia is ~4 g/day [5]. Although a randomized controlled CVD outcomes trial evaluating ~4-g per day of ethyl eicosapentaenoic acid (EPA) in addition to baseline statin therapy is underway, results are not expected until 2017 [6].

Pending results of CVD outcome trials, an assessment of the effects of omega-3 fatty acids on preclinical predictors of CVD events may be informative. Omega-3 fatty acids reduce inflamation [7], but improvements in pulse wave velocity (PWV), have not been demonstrated. This may reflect inadequate dosing or a lower baseline CVD risk among populations studied. For example, despite 12-months of therapy with 1.8 g of omega-3 fatty acids daily, no reduction in arterial stiffness was detected among overweight but otherwise healthy middle aged subjects [8], suggesting that both treatment intensity as well as underlying CVD risk profile may be important determinants of benefit.

Arterial PWV is an independent predictor of CVD events among patients with hypertension [9] and is the gold standard measurement of arterial stiffness [10]. Given our previous observation that PWV was an independent predictor of preclinical atherosclerosis [11], we assessed effect of high-dose (3.36 g) omega-3 fatty acids on PWV and secondarily high sensitivity C-reactive protein (hsCRP), lipoprotein-associated phospholipase A2 (Lp-PLA2), and serum adiponectin.

## Methods

We conducted a prospective, randomized placebo-controlled, double-blind pilot study. Patients received either 4 omega-3 fatty acids (Lovaza™ Glaxo Smith Kline, United Kingdom) capsules or identically matched corn-oil placebo. Each Lovaza capsule includes 465 mg of EPA and 375 mg of docosahexaenoic acid (DHA) for a total daily dose of 3.36 g. The treatment period was 3-months with baseline and follow-up measurements performed in the morning in a fasted state. The Colorado Multiple Institutional Review Board approved the study and it was registered with clinicaltrials.gov. All study participants signed written informed consent.

Subjects were recruited from outpatient primary care clinics or a preexisting registry of hypertension patients. This cohort consisted of 177 individuals; the inclusion and exclusion criteria for this registry have been previously reported [11]. Eligible patients were ≥18 years of age, of either Latino or non-Latino White ethnicity and had at least one other CVD risk factor including diabetes, dyslipidemia, obesity, chronic kidney disease, microalbuminuria, current smoking, or age >55 for men or >65 for women, but were excluded if they had pre-existing CVD.

Arterial PWV measurements were performed in the recumbent position. Supine blood pressure was measured in duplicate in the non-dominant arm. Bilateral brachial-ankle PWV was derived from the pulse transit time between and the estimated path length between proximal and distal arterial sites expressed as cm/s. Inflammatory markers and adiponectin were also assessed while fasting. We chose hsCRP because it has incremental CVD risk discrimination beyond standard Framingham risk factors [12] and Lp-PLA2 given its specificity for inflammation localized to atherosclerotic plaque [13].

### Statistical analysis

Means, standard deviations, and medians were calculated for all continuous variables. For univariate analyses, comparisons were made using analysis of variance, chi-squared or Wilcoxon rank sum tests. For change over time analyses, mixed-effects models were used to account for repeated measures within participants. Univariate associations between baseline risk markers and change in PWV were assessed, and multivariate models were fitted to assess for predictors of change. Sensitivity analyses were performed to assess changes in outcomes among the following subgroups: patients naïve to statin therapy, those with baseline systolic blood pressure ≥ 140 mm Hg, and diabetic patients, *P*-values <0.05 were considered statistically significant. SAS Version 9.4 (Cary, NC) was used for all statistical analyses.

## Results

Baseline characteristics of the 62 participants are shown in Table 1 and were consistent with a safety-net population. The majority of patients were receiving medication for chronic hypertension and half had diabetes. Overall, baseline characteristics were well matched; specifically, PWV values did not differ by randomization group. Among baseline variables, older age, higher systolic blood pressure, and adiponectin were significantly associated with increased PWV: 16 cm/s increase in mean PWV per year of increasing age (*p* < .0001), 7.3 cm/s increase in mean PWV per each mm Hg of higher systolic blood pressure (*p* = 0.005), and 14 cm/s per unit of adiponectin (*p* = 0.008).

**Table 1:** Baseline sociodemographic and clinical characteristics

		Placebo (*N* = 35) N (%) or Mean (SD)	Omega-3 (*N* = 27) N (%) or Mean (SD)	Overall (*N* = 62) N (%) or Mean (SD)
Age (years)		60.2 (10.8)	62.3 (9.7)	61.1 (10.3)
Female Gender		22 (63 %)	18 (67 %)	40 (65 %)
Race/ethnicity	Non-Latino White	7 (20 %)	11 (41 %)	18 (29 %)
	Latino	28 (80 %)	16 (59 %)	44 (71 %)
Educational Status	Did Not Complete High School	18 (51 %)	10 (37 %)	28 (45 %)
	Completed High School	9 (26 %)	10 (37 %)	19 (31 %)
	Completed College	8 (23 %)	7 (26 %)	15 (24 %)
Unemployed		28 (80 %)	21 (81 %)	49 (80 %)
Body Mass Index (kg/m^2^)		31.5 (7.1)	33.9 (8.6)	32.6 (7.8)
Systolic Blood Pressure (mm Hg)^a^		137 (16)	128 (14)	133 (16)
Diastolic Blood Pressure (mm Hg)		82 (9)	78 (10)	81 (10)
Antihypertensive Medication		32 (91 %)	21 (78 %)	53 (85 %)
Statin therapy		17 (49 %)	11 (41 %)	28 (45 %)
Total cholesterol (mg/dL)		179 (43)	179 (38)	179 (40)
Triglycerides (mg/dL)		188 (103)	173 (65)	182 (89)
HDL-C (mg/dL)		48.4 (14.9)	44.9 (12.4)	46.9 (13.9)
LDL-C (mg/dL)		97 (38)	99 (29)	98 (34)
Diabetes diagnosis		19 (54 %)	12 (48 %)	31 (52 %)
Hemoglobin A1c (%)		6.7 (1.8)	6.3 (1.3)	6.6 (1.6)
Glucose (mg/dL)		127 (61)	111 (25)	120 (49)
Smoking status	Current	12 (34 %)	6 (22 %)	18 (29 %)
	Former	23 (66 %)	21 (78 %)	44 (71 %)
hsCRP (mg/L)		3.42 (3.35)	5.63 (5.05)	4.38 (4.29)
Adiponectin (ug/mL)		10.6 (8.3)	12.2 (7.8)	11.4 (8.1)
LpPLA2 mass (ng/mL)		244 (46)	252 (62)	247 (53)
Mean PWV (cm/s)		1690 (335)	1602 (324)	1652 (330)

*PWV* pulse wave velocity, *CRP* C-reactive protein, *Lp-PLA2* lipoprotein-associated phospholipase A2

^a^Systolic Blood Pressure difference between groups at *p* < 0.05 using Wilcoxon rank sum test

**Fig. 1:**
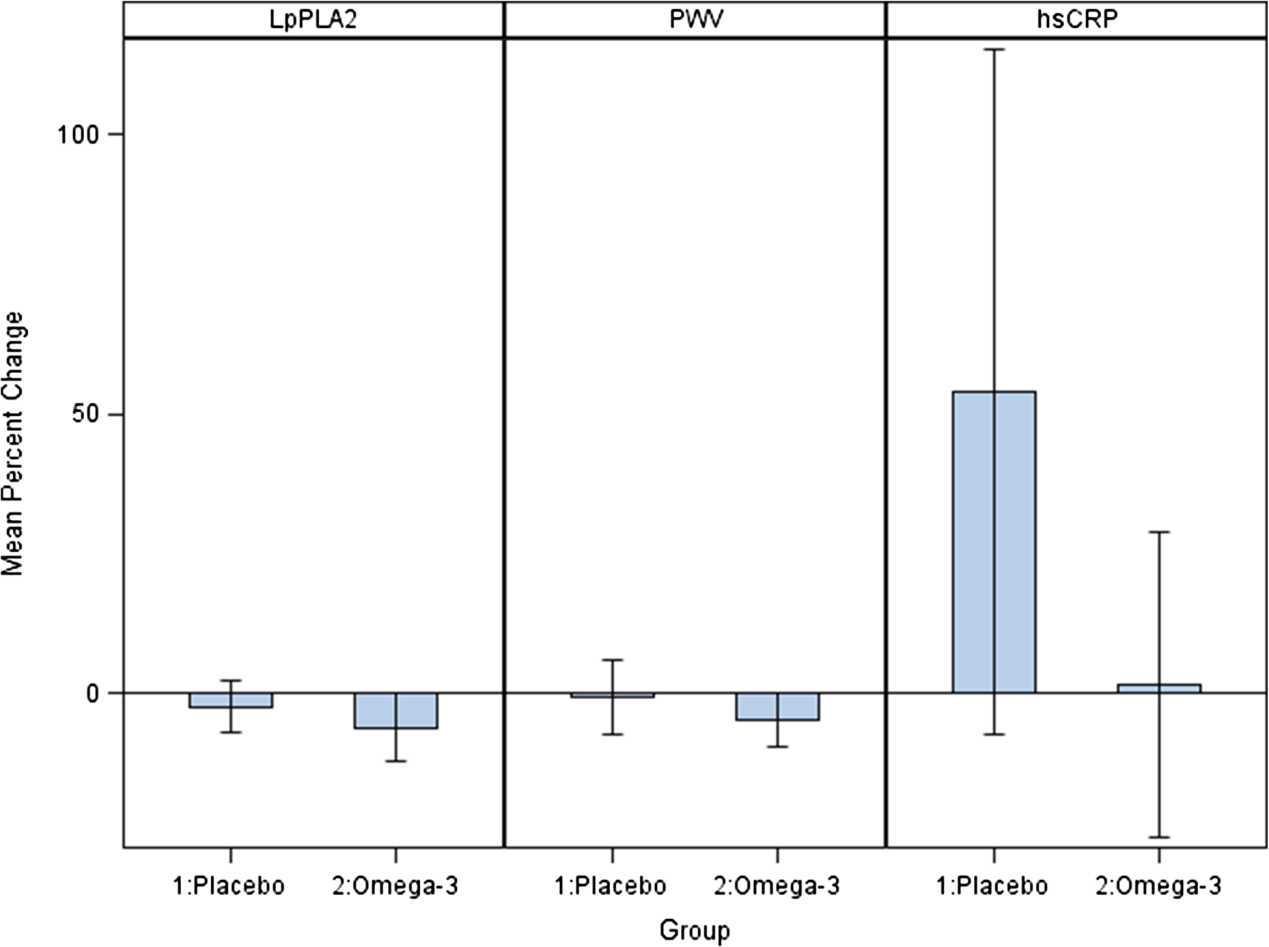
Mean change in arterial pulse wave velocity (PWV) from baseline to 3-months based on randomized treatment assignment

Changes in risk factors, inflammatory markers, and PWV are shown in Table 2. Comparative percentage change in Lp-PLA2 mass, PWV, and hsCRP were all directionally more favorable in the omega-3 arm but did not achieve statistical significance (Fig. 1). Absolute change in mean PWV was −97 cm/s in the omega-3 arm compared to −33 cm/s in the placebo group (*p* = 0.36). Reductions were also seen in mean hsCRP (−0.9 mg/L vs. 0.9 mg/L in placebo group) and Lp-PLA2 mass (−18.1 ng/mL vs. −6.1 ng/mL). Numeric mean reductions in risk markers were relatively larger within subgroups: Among 34 statin-naïve subjects, the difference in arterial PWV was larger (−82 vs. +50 cm/s), but remained non-significant (*p* = 0.20), though the reduction in mean hsCRP (− 0.8 vs. +1.6 mg/dl) achieved significance (*p* = 0.03). Among 31 diabetic subjects, PWV (−100 vs. −18 cm/s), hsCRP (−0.8 vs. +1.7 mg/L), and LpPLA-2 mass (−11.1 vs. −4.1 ng/ml) were non-significantly lower with active treatment (minimal *p*-value 0.19). Among 24 subjects with baseline systolic blood pressure ≥ 140 mm Hg PWV (−98 vs. −65 cm/s), hsCRP (−1.0 vs. +0.8 mg/L), and LpPLA-2 mass (−32.7 vs. −3.2 ng/ml) were non-significantly lower with active treatment (minimal *p*-value 0.09).

**Table 2:** Change in risk marker values from baseline to 3-months

	Placebo (*N* = 35) Mean (SD)	Omega-3 (*N* = 27) Mean (SD)
Pulse Wave Velocity (cm/s)	−33 (306)	−97 (182)
Total cholesterol (mg/dL)	−6.6 (30.4)	−0.8 (18.1)
Triglycerides (mg/dL)	−30.0 (58.1)	−17.6 (45.6)
HDL-C (mg/dL)	0.2 (8.5)	2.9 (14.6)
LDL-C (mg/dL)	−2.8 (28.6)	0.7 (18.3)
Hemoglobin A1c (%)	−0.13 (0.94)	0.06 (0.44)
Glucose (mg/dL)	−13.1 (44.0)	0.6 (23.0)
hsCRP (mg/L)	0.9 (4.4)	−0.9 (3.1)
Adiponectin (ug/mL)	0.3 (3.4)	−0.4 (2.4)
LpPLA2 mass (ng/mL)	−6.1 (31.7)	−18.1 (41.1)

*CRP* C-reactive protein, *Lp-PLA2* lipoprotein-associated phospholipase A2

In multivariate analysis accounting for baseline age, systolic blood pressure and adiponectin, no significant change in mean PWV [parameter estimate (standard error) = -22 (24), *p* = 0.36] was observed. In analysis including only time and treatment group, the reductions in hsCRP and Lp-PLA2 mass were numerically greater with omega-3 therapy, but were not statistically significant (*p* = 0.08, and 0.21, respectively).

## Discussion

To our knowledge, this is the first prospective randomized trial evaluating the effects of prescription doses of omega-3 fatty acids on arterial stiffness in a Latino-predominant population. Short-term treatment with omega-3 fatty acids was not associated with a significant reduction in arterial PWV. Moreover, with the exception of a reduction in serum hsCRP among statin-naïve subjects, no significant improvements in markers of vascular inflammation were observed despite a high prevalence of obesity and diabetes. Given an association between the metabolic syndrome and increased arterial stiffness [14], a positive effect of omega-3 fatty acids might have been expected.

A number of possible explanations for our findings merit consideration. One potentially important factor is the dose of omega-3 utilized. In one study, PWV was assessed among overweight patients receiving 2, 4, and 6-g of omega-3 fatty acids daily [15]. Reductions in PWV were observed only in the group receiving 6-g per day. It is possible that despite the 3.36 g dose in the current study, it was still inadequate to reduce PWV, particularly if compliance was sub-optimal. Although no medication diary or formalized drug reconciliation process was utilized in our study, this is plausible given the absence of a significant triglyceride reduction observed in the active treatment arm, which may reflect medication non-adherence in our vulnerable population. Also, half of the patients in our study were already receiving statin therapy, which could limit our ability to further discern a treatment effect. In support of this possibility, a recent trial among patients with peripheral arterial disease already receiving statin therapy, found no improvement in PWV after omega-3 fatty acid treatment [16]. Our findings are in line with this possibility since the difference in PWV over time between the groups was larger among statin-naïve subjects. Analogously, an expected greater reduction in hsCRP was seen among statin-naïve subjects. One further study limitation is that fatty acid bioavailability data were not evaluated, so we don’t know if there was a relationship between plasma fatty acid level and changes in PWV.

Another potential explanation for the findings in the current study is the relatively small sample size. Root and colleagues also found no reduction in PWV with omega-3 therapy in a short-term study of 57 patients [17]. In assessing sample size, approximately 100 subjects would provide > 80 % power to detect a 10 % decrease in PWV (standard deviation [SD] 350 cm/s) assuming a baseline PWV of 1700 cm/s. With 62 randomized patients, the current study had just over 60 % power under those assumptions. Although the numeric effect size in the current trial was consistent with this reduction, and the standard deviation was within assumed range, the placebo-corrected absolute reduction in PWV was only 4 %. The clinical significance of this numeric finding may be gleaned from a meta-analysis of observational data from 17,635 subjects, where a 10 % increase in PWV was associated with a hazard ratio for CVD events of 1.07 (95 % CI: 1.02 to 1.12) [18].

In addition, the relatively short duration of therapeutic exposure, may have limited our ability to detect alterations in vascular stiffness. There was also a numeric imbalance of subjects between arms, which likely reflects a chance finding in the randomization sequence given the small sample size. This theoretically may have made a Type II error more likely. Most importantly, however, no pharmacologic therapy to date has been shown to reduce PWV independent of blood pressure reductions, suggesting the possibility that any salutatory effects of omega-3 treatment on inflammation and plaque may be inadequate to alter vessel wall physiology.

## Conclusions

In conclusion, high-dose purified omega-3 fatty acids did not significantly improve arterial stiffness among hypertensive patients extending the negative results of previous studies. Given the absence of benefits of omega-3 fatty acids on CVD events in large randomized controlled clinical trials, this therapy cannot be uniformly recommended in primary prevention patients despite widespread use.

## Abbreviations

CVD: cardiovascular disease
EPA: eicosapentaenoic acid
hsCRP: high sensitivity c-reactive protein
LDL: low density lipoprotein
Lp-PLA2: lipoprotein-associated phospholipase A2
LUCHAR: latinos using cardio actions to reduce risk
PWV: pulse wave velocity
SD: standard deviation
SE: standard error
